# *BJS Open* 2024 best colorectal surgery articles: editors’ choices

**DOI:** 10.1093/bjsopen/zraf005

**Published:** 2025-03-10

This article highlights some of the most impactful colorectal studies published in *BJS Open* in 2024.

The first is a nationwide study^1^ that focused on the incidence of rectal cancer after subtotal colectomy in patients with inflammatory bowel disease (IBD). It included 8120 patients who underwent subtotal colectomy and a total of 61 patients (0.8%) were diagnosed with rectal cancer, giving rise to a cumulative incidence of 0.26%, 0.49%, and 0.77% at 5, 10, and 15 years respectively. This study successfully highlighted that the incidence of rectal cancer after subtotal colectomy is low. Factors associated with an increased incidence of rectal cancer were identified, including prior diagnoses of colonic dysplasia and primary sclerosing cholangitis. Notably, it also demonstrated a significant decline of 43% in endoscopic surveillance procedures during 2020, likely due to the COVID-19 pandemic.

The next is a European Society of Coloproctology study that assessed risk factors associated with anastomotic leaks after circular stapled left-sided bowel surgery^2^. Among the 3305 procedures analysed, the anastomotic leak rate was 8.0% and 2.1% of patients had an unplanned intensive care unit (ICU) stay. The study identified critical factors that predict anastomotic leaks, including male sex, conversion from minimally invasive to open surgery, and the height of the anastomosis, specifically for operations involving the lower third of the rectum. However, there were no variations in the rates of anastomotic leaks and unplanned ICU stays based on stapler head diameter, geographical location, or surgeon experience. While the choice of the circular stapler brand was deemed inconsequential to leak rates, the research highlights that surgical technique and patient characteristics remain critical to patient outcomes.

Another study evaluated the efficacy of hyperthermic intraperitoneal chemotherapy (HIPEC) in colorectal cancer in a cohort of 2093 patients treated between 1991 and 2018^3^. The study documented that oxaliplatin-HIPEC demonstrated significantly improved overall survival outcomes compared with mitomycin-HIPEC (median overall survival of 47 *versus* 39 months) and the difference in 90-day mortality favoured the oxaliplatin-HIPEC group. High-dose mitomycin-HIPEC showed comparable results to oxaliplatin-HIPEC. Additionally, a trend indicating a dose–response relationship between low- and high-dose HIPEC was observed. Beyond survival outcomes, the study also assessed the postoperative morbidity associated with both regimens.

In addition to colorectal cancer papers, *BJS Open* has published papers that highlight innovations in surgery to improve patient outcomes. These innovations include continuous monitoring of vital signs (CMVS) and wearable wireless devices that can facilitate early detection of clinical deterioration^4^. A total of 908 patients were included in this study and the median length of hospital stay was lower in the CMVS group (5.0 *versus* 5.5 days; *P* = 0.012). There was a decrease in calls from nursing staff to medical teams (from 15.3% to 7.7%; *P* = 0.007) but an increase in nursing activities in the CMVS group. However, all secondary clinical outcomes were similar in the CMVS and control groups. This study highlighted the potential increased use of smart technologies to increase ambulatory care and closer less invasive monitoring of patients.

Finally, a comprehensive review^5^ evaluated childhood conditions, including atresia, tracheo-oesophageal fistula, malrotation, short bowel syndrome, gastroschisis, exomphalos, choledochal malformations, Hirschsprung disease, anorectal malformations, congenital diaphragmatic hernia, congenital lung lesions, and paediatric cancer, and their lifelong implications. It highlighted the increasing survivorship of children affected by surgical conditions, translating into an increasing number of adults with lifelong conditions and specialist healthcare needs. This paper aimed to promote systems to offer care to this potentially vulnerable group and the importance of collaborating with paediatric surgeons in providing successful and seamless transitional care.

The studies published in *BJS Open* in 2024 showcase the dynamic colorectal research-driven advancements in the field. Continued exploration in these areas is essential for enhancing surgical outcomes and patient safety across diverse clinical practices and the editors thank the authors of the study manuscripts for trusting *BJS Open* for their research.
